# Application of depithelized gracilis adipofascial flap for pelvic floor reconstruction after pelvic exenteration

**DOI:** 10.1186/s12893-022-01755-0

**Published:** 2022-08-06

**Authors:** Chen Zhang, Xin Yang, Hongsen Bi

**Affiliations:** Department of Plastic Surgery, Peking University Third Hospital, 49 North Garden Road, Haidian District, Beijing, 100191 China

**Keywords:** Pelvic exenteration, Pelvic floor reconstruction, Gracilis flap, Adipofascial flap

## Abstract

**Background:**

Pelvic exenteration is a radical surgery performed in selected patients with locally advanced or recurrent pelvic malignancy. It involves radical en bloc resection of the adjacent anatomical structures affected by the tumor. The authors sought to evaluate the clinical application of a depithelized gracilis adipofascial flap for pelvic floor reconstruction after pelvic exenteration.

**Methods:**

A total of 31 patients who underwent pelvic floor reconstruction with a gracilis adipofascial flap after pelvic exenterationat Peking University Third Hospital from 2014 to 2022 were enrolled in the study. The postoperative follow-up durations varied from 4 to 12 months.

**Results:**

The survival rate of the flap was 96.77% with partial flap necrosis in one case. The total incidence of postoperative complications associated with the flap was 25.81%, with an incidence of 6.45% in the donor site and 19.35% in the recipient site. All complications were early complications, including postoperative infection and flap necrosis. All patients recovered after treatments, including anti-infectives, dressing change, debridement, and local flap repair. Long-term follow-up showed good outcomes without flap-related complications.

**Conclusions:**

A depithelized gracilis adipofascial flap can be applied for pelvic floor reconstruction after pelvic exenteration. The flap is an ideal and reliable choice for pelvic floor reconstruction with few complications, an elevated survival rate, sufficient volume, and mild effects on the function of the donor site.

## Introduction

Pelvic exenteration involves radical en bloc resection of the adjacent anatomical structures affected by a tumor. The procedure was first described in 1948 by Alexander Brunschwig and applied in the treatment of pelvic malignancy. According to the site of recurrence, pelvic exenteration mainly comprises three types: (1) Anterior pelvic exenteration for tumors affecting the bladder and urethra requiring resection of the entire bladder (including urethra), uterus, prostate, and vagina are resected; (2) posterior pelvic exenteration for tumors affecting the rectum, in which the vagina, uterus prostate, and affected rectum are resected; and (3) total pelvic exenteration for tumors affecting the bladder and rectum, in which the bladder (including urethra), vagina, uterus, prostate, and rectum are resected. Based on the resection area, pelvic exenteration is classified into three types: type I involves the resection margin above the levator ani; type II is the resection area that includes the levator ani; and type III is the resection margin extending below the levator ani [1, 2].

Previous studies reported rates of intraoperative, early postoperative, and long-term complications of ∼ 30%, suggesting that pelvic exenteration is an effective surgical measure to achieve complete resection for a majority of patients [3, 4].

Patients who underwent pelvic exenteration have a low 5-year survival rate according to early literature. Along with the confirmation of surgical indications, mastery of surgical techniques, and improved diagnosis and treatment for perioperative complications, the postoperative 5-year survival rate has increased from 20% in an earlier period to 64% [5]. A systematic review indicated that a positive surgical margin is an important prognostic indicator in pelvic exenteration. However, presurgical evaluations through magnetic resonance imaging and positron emission tomography-computed tomography cannot predict the surgical margin condition. Therefore, the author proposed that radical exenteration (type III exenteration) may achieve a more negative margin result [6]. In patients undergoing type III pelvic exenteration, a large area of perineal skin and subcutaneous tissues is missing, thereby preventing the perineum from closing and requiring flaps for perineal reconstruction.

Pelvic exenteration is a complicated procedure with intraoperative and postoperative comorbidity-related mortality rate of 0–12%. Previous studies have shown that the survival rates of patients with postoperative complications is significantly reduced [5]. Resection of pelvic tissues that receive radiotherapy may lead to various fistulas, poor wound prognosis, and secondary problems caused by ureteral or intestinal obstruction. However, type III exenteration could reduce the incidence of complications [7], such as intestinal obstruction, pelvic abscess, and fistula formation [8–10], in patients who need pelvic floor reconstruction.

Therefore, pelvic floor reconstruction after type III exenteration is crucial to the success of the entire treatment. At present, the most popular technique for pelvic floor reconstruction is the application of rectus abdominis flaps, including the vertical rectus abdominis flap, transverse rectus abdominis flap, and deep inferior epigastric perforator flap [11–15]. However, abdominal flaps will compromise the integrity of the abdominal wall, resulting in a weak abdominal wall, asymmetric abdominal wall contour, and abdominal hernia. It may also cause a series of flap-related complications. In addition, many patients undergoing pelvic exenteration require urethrostomy and/or enterostomy. The incomplete abdominal wall will limit the choice of stoma location and increase the difficulty of ostomy [16]. The gracilis flap is currently used to repair perineal defects, wounds, or fistulas. Compared with the rectus abdominis, the gracilis has many synergistic muscles. Harvesting the gracilis will lead to mild effects on thigh function. The muscle flap has normal tensile resistance and tension with long blood vessels and nerve pedicles in a superficial anatomical position. Hence, it is simple and feasible to harvest, which makes it an ideal donor muscle [17].

From July 2014 to January 2022, we performed pelvic floor reconstruction with unilateral or bilateral depithelized gracilis adipofascial flaps in 31 postexenteration patients and achieved good outcomes. In this article, we aimed to describe the method and review the flap surgical outcomes in these patients.

## Clinical data

This was a retrospective cohort study of 31 patients undergoing pelvic floor reconstruction with gracilis flaps after pelvic exenteration in our hospital from July 2014 to January 2022. Pelvic exenteration was decided and performed by gynecologists for oncological indication. The following data were obtained: patient demographics, operative details, postoperative early (< 30 days) and long-term (> 30 days) complications associated with the flaps. Complications were classified according to the Clavien–Dindo classification. This study was approved by the Ethics Committee of Peking University Third Hospital (Table 1).

**Table 1:** Patient details and complications associated with the flap application

	Cases (Total: 31)	Percentage
Diagnosis		
Cervical cancer	22	70.97%
Vaginal cancer	5	16.13%
Endometrial cancer	4	12.90%
Flap		
Unilateral	20	64.51%
Bilateral	11	35.48%
Complications associated with the flaps		
Early		
Total	8	25.81%
Clavien–Dindo grade II	3	9.68%
Clavien–Dindo grade IIIa	4	12.90%
Clavien–Dindo grade IIIb	1	3.23%
Long-term	0	0
Donor site		
Total	2	6.45%
Clavien–Dindo grade II	1	3.23%
Clavien–Dindo grade IIIa	1	3.23%
Recipient site		
Total	6	19.35%
Clavien–Dindo grade II	2	6.45%
Clavien–Dindo grade IIIa	3	9.68%
Clavien–Dindo grade IIIb	1	3.23%

## Surgical methods

All surgeries were performed under general anesthesia in the lithotomy position. Total pelvic exenteration was performed by gynecologists, while urethrostomy was performed by urologists, in which four patients underwent Bricker Conduit. Enterostomy was performed by general surgeons.

**Fig. 1:**
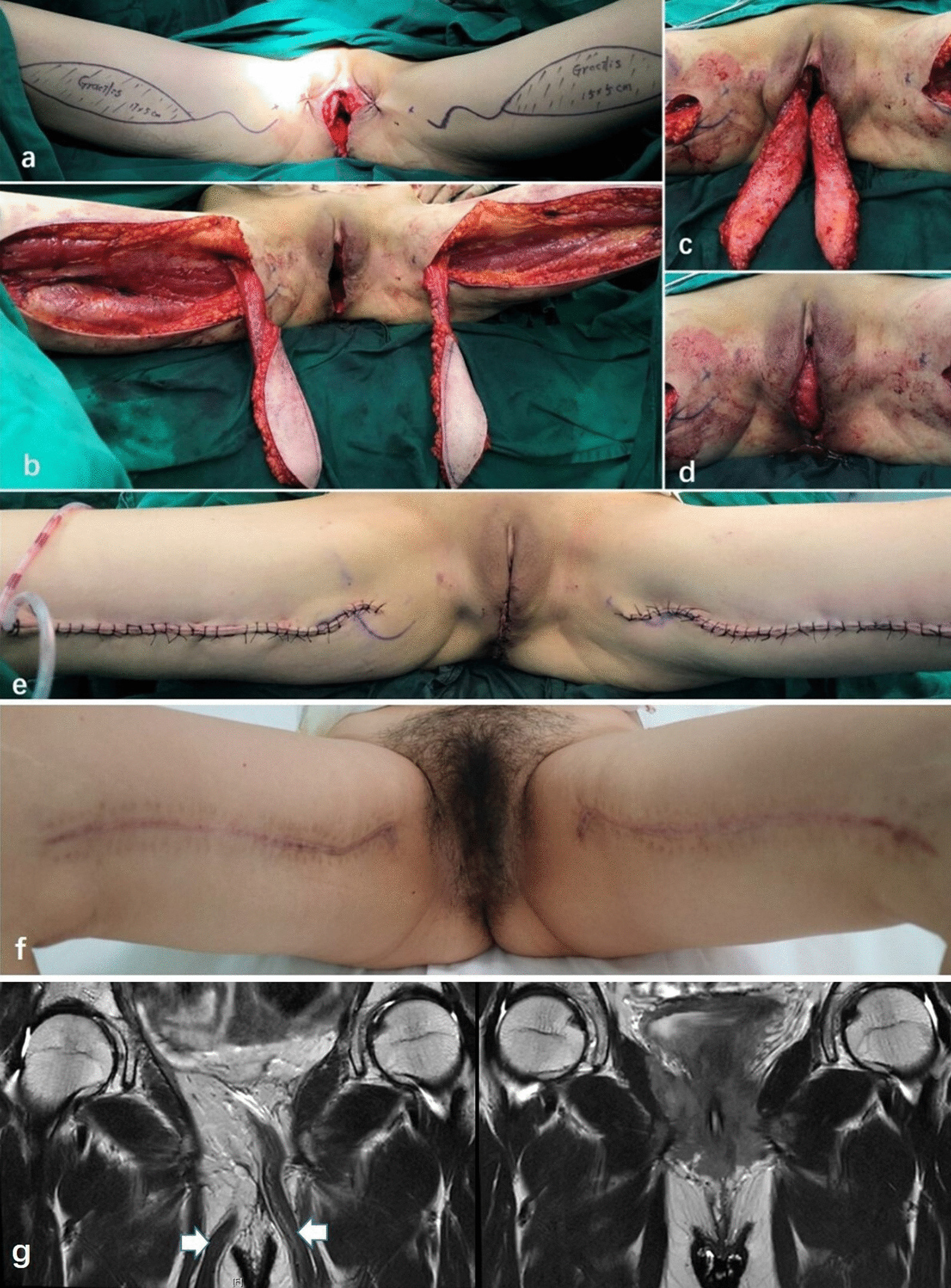
**a** Pelvic floor defect after pelvic exenteration and design of gracilis flaps on the inner side of both thighs. **b** The flap is freed with the proximal end of the muscle as a pedicle. **c** The skin was removed, and the muscle and subcutaneous tissues of the fascia were retained to prepare the gracilis adipofascial flap. The flap was rotated at 180° into the pelvic floor defect through the subcutaneous tunnel. **d** The pelvic floor defect was repaired with the gracilis adipofascial flap. **e** The recipient and donor sites were sutured. **f** 8 months postoperatively. **g** Gynecological MRI indicated good survival of the muscle flap, as marked by the white arrow (preoperative on the left side and postoperative 4 months on the right side)

The gracilis flaps were harvested by plastic surgeons. The incision line was designed along the long axis of the gracilis on the inner thigh. The anterior and posterior edges of the flap are usually located approximately 3–4 cm outside the edge of the gracilis with the proximal end in the gluteal fold and the distal end on the inner side approximately 5 cm above the upper end of the medial tibial condyle. The skin and subcutaneous tissues were incised on the inner side of the knee joint at the distal end of the flap along the incision line, and the great saphenous vein, the sartorius, and the gracilis tendon, which were on the deep side of the sartorius, were explored. The gracilis tendon was dissected, and the flap direction was reconfirmed. The skin was cut along the incision line, and the subcutaneous tissues were dissected obliquely downward to expose the deep fascia of the gracilis. The gracilis was carefully dissected along the deep fascia with special attention to protect the perforating vessels at the proximal end of the muscle. Approximately whole muscle used for the flap. The myocutaneous flap was then freed from the distal side to the proximal side. The skin was removed, and the muscle with the subcutaneous tissues of the fascia was retained to prepare the adipofascial flap. The distal soft tissue with poor blood supply was removed untill the blood supply of the distal end of the flap was good. The subcutaneous tunnel and muscle pedicle were formed by dissection subcutaneously from the proximal end of the gracilis adipofascial flap toward the perineum. The flap was rotated along the pedicle at 180° into the pelvic floor defect through the subcutaneous tunnel. The drainage tube was placed in the recipient site, and the advanced flaps were freed subcutaneously on both sides of the perineal incision. The wound was closed by tension-reduced suture. The donor site was stretched and sutured and placed under vacuum drainage. If the pelvic floor defect was large, bilateral gralicis adipofascial flaps were applied, which were inset in the defect in parallel. (Fig. 1).

The drainage tubes were removed when the postoperative drainage was less than 20 ml, generally within 10 days after surgery. Usually, the patient could exercise on the floor on the ward 2 days after surgery, and excessive and prolonged hip flexion on the ipsilateral side should be avoided within 1 week.

## Results

The patients were 31 to 68 years old, with an average age of 53.87 ± 9.70 years. Twenty two cases were cervical cancers, four cases were endometrial cancers, and five cases were vaginal cancers. Twenty patients underwent unilateral gracilis surgery, and the 11 other patients underwent bilateral surgery. None of the patients selected to undergo vaginal reconstruction. The patients were followed up for 4–12 months after surgery.

The survival rate of the flap was 96.77%, with partial flap necrosis in one case. We further performed surgery to remove the necrotic tissues and repair the partial perineal defect by translocation of the local flap on the 23rd postoperative day.

The total incidence of postoperative flap-related complications was 25.81%. The incidence of complications in the donor site was 6.45%, in which two patients developed donor site infection. One was Clavien–Dindo (CD) grade II and one was CD grade IIIa. After anti-infective and dressing changes, one patient showed improvement, while another patient underwent debridement and suturing on the 21st postoperative day due to poor infection control. The incidence of complications in the recipient site was 19.35%. Three were CD grade II, four were CD grade IIIa, and one was CD grade IIIb. Four patients developed infections in the perineal area. After anti-infective and dressing changes, two patients showed improvement, while two underwent debridement and suture on the 17th and 36th postoperative days respectively due to poor control. One patient who developed infection had poor control after dressing and treatment with antibiotics, resulting in perineal incision dehiscence, requiring debridement and suture on the 22nd postoperative day. Another patient developed partial flap necrosis and incision dehiscence, and a secondary operation was performed as previously described. Long-term follow-up showed good outcomes without flap-related complications (Table 1).

## Discussion

The gracilis is located subcutaneously on the inner side of the thigh and belongs to the adductor muscle group. It is a long, flat, and belt-shaped muscle that starts from the anterior of the pubic and ischial rami and ends at the medial side of the upper end of the tibia, with an average length of 25–44 cm and an average width of 5–7 cm. The main nourishing vessel, 73–87% of which originates from the deep femoral artery, runs between the adductor magnus and the adductor brevis, with two accompanying veins and an obliquely inward blood vascular pedicle. The blood vessel enters the muscle at the proximal one-third and 8–13 cm away from the pubic symphysis on the deep side. The length of the vascular pedicle is 5.5–11 cm, and the outer diameters of the vessel are 0.5–0.8 mm at the muscle hilum and 1.5–2.0 mm near the proximal femoral artery. The gracilis is innervated by the anterior branch of the obturator nerve, which runs obliquely inward and downward along the deep side of the adductor longus and divides five to seven branches into the muscle at the junction of the upper and the middle one-third of the gracilis [17–19].

Harvesting the gracilis flap has only a mild effect on the function of the lower extremity because of sufficient synergistic muscles, thereby making the flap an ideal graft donor. The gracilis was first applied to repair the anal sphincter in children in 1952 [20]. In 1976, Harii et al. [21] used free gracilis muscles to repair facial paralysis in two cases. At present, only a few studies have reported on the application of gracilis muscles for pelvic floor reconstruction [16, 22–34]. The sartorius runs obliquely through the superficial side of the distal part of the gracilis. Since there is no myocutaneous artery branching into the skin in this segment of the gracilis, distal necrosis of the musculocutaneous flap may occur [18, 19, 35]. Yousif et al. [36] reported that using the proximal one-third of the transverse gracilis flap reduces the incidence of distal necrosis compared with the vertical muscular flap. Kaartinen et al. [37, 38] applied this procedure to pelvic floor reconstruction and vaginal reconstruction after pelvic exenteration, achieving good outcomes. Singh et al. [16] reported the use of the gracilis muscular flap to repair the pelvic cavity and completed the tension-reduced suture of local perineal incisions through VY or advanced flaps, thereby reducing the incidence of postoperative necrosis.

However, compared with abdominal flaps, such as the vertical rectus abdominis flap, the transverse rectus abdominis flap, and the deep inferior epigastric perforator flap, both the transverse gracilis and the gracilis muscular flaps have insufficient volume. Therefore, we used the depithelized gracilis adipofascial flaps for pelvic floor reconstruction after pelvic exenteration, which considerably increased the tissue supply and facilitated the repair of postexenteration defects. The tissue defect in the recipient site was repaired using advanced flaps that were freed on both sides of the incision subcutaneously to achieve tension-reduced suture.

Pelvic floor reconstruction via the depithelized gracilis adipofascial flap has certain advantages compared with that via flaps after pelvic exenteration. The gracilis adipofascial flap prevents complications, such as a weak abdominal wall, asymmetric abdominal wall contour, and abdominal hernia, compared with the the abdominal flaps, as the former maintains the integrity of the abdominal wall. In addition, performing rectus abdominis flap surgery among patients who undergo abdominal wall incision has increased difficulty due to damage to the rectus abdominis. Anterolateral thigh flap (ALT) is also an option [39]. But compared with gracilis flap, more soft tissues are wasted in the process of transferring ALT flap to the medial side of the thigh, resulting in limited pelvic reconstructive volume. In addition, a scar in the donor part in medial thigh considered to be more conceal. In this case, pelvic floor reconstruction with gracilis flaps is the better choice. Compared with the use of the traditional vertical gracilis musculocutaneous flap, the depithelized gracilis adipofascial flap technique reduces the incidence of distal flap necrosis. In the 31 cases that we treated, the survival rate of the flap was 96.77% and the incidence of early postoperative flap-related complications was 25.81%, of which the incidence in the donor site was 6.45%, and the incidence in the recipient site was 19.35%. None of the patients with early postoperative complications developed long-term complications. The incidence of complications was not high compared with that in previous studies [11, 40–42]. Compared with the aforementioned transverse gracilis flap and the gracilis muscular flap, the gracilis adipofascial flap has a larger volume and can be used for the pelvic floor reconstruction of larger postexenteration defects. However, this technique has several limitations. It cannot be used for vaginal reconstruction, thus it is only suitable for patients who do not require vaginal reconstruction. Additionally, a large skin defect in the perineum after pelvic exenteration cannot be repaired by VY or local flaps. The flaps with skins should still be given priority to perform pelvic reconstruction. In addition, according to our experience, the success of the depithelized gracilis adipofascial flap has a certain height requirement; that is, a shorter stature results in less tissue of the gracilis flap. In this case, priority should be given to the rectus abdominis flap.

## Conclusion

The depithelized gracilis adipofascial flap can be used for pelvic floor reconstruction after pelvic exenteration. This technique results in few complications, an elevated survival rate, sufficient volume, and mild effects on the function of the donor site. As such, we believe that the depithelized gracilis adipofascial flap is an ideal and reliable choice for pelvic floor reconstruction.

## Data Availability

The datasets of the current study are available from the corresponding author upon reasonable request.
